# Inhibition of Phosphatase and Tensin Homolog Deleted on Chromosome 10 Decreases Rat Cortical Neuron Injury and Blood-Brain Barrier Permeability, and Improves Neurological Functional Recovery in Traumatic Brain Injury Model

**DOI:** 10.1371/journal.pone.0080429

**Published:** 2013-11-28

**Authors:** Jun Ding, Jianyi Guo, Qiang Yuan, Fang Yuan, Hao Chen, Hengli Tian

**Affiliations:** Department of Neurosurgery, Shanghai 6 ^th^ People's Hospital, Shanghai Jiaotong University, Shanghai, China; University of South Florida, United States of America

## Abstract

**Background and Purpose:**

Recent evidence has supported the neuroprotective effect of bpV (pic), an inhibitor of phosphatase and tensin homolog deleted on chromosome 10 (PTEN), in models of ischemic stroke. However, whether PTEN inhibitors improve long-term functional recovery after traumatic brain injury (TBI) and whether PTEN affects blood brain barrier (BBB) permeability need further elucidation. The present study was performed to address these issues.

**Methods:**

Adult Sprague-Dawley rats were subjected to fluid percussion injury (FPI) after treatment with a well-established PTEN inhibitor bpV (pic) or saline starting 24 h before FPI. Western blotting, real-time quantitative PCR, or immunostaining was used to measure PTEN, p-Akt, or MMP-9 expression. We determined the presence of neuron apoptosis by TUNEL assay. Evans Blue dye extravasation was measured to evaluate the extent of BBB disruption. Functional recovery was assessed by the neurological severity score (NSS), and Kaplan-Meier analysis was used for survival analysis.

**Results:**

PTEN expression was up-regulated after TBI. After bpV (pic) treatment, p-Akt was also up-regulated. We found that bpV (pic) significantly decreased BBB permeability and reduced the number of TUNEL-positive cells. We further demonstrated that PTEN inhibition improved neurological function recovery in the early stage after TBI.

**Conclusion:**

These data suggest that treatment with the PTEN inhibitor bpV (pic) has a neuroprotective effect in TBI rats.

## Introduction

Traumatic brain injury (TBI) is a leading cause of morbidity and disability in modern society, especially in young people. Neurological function impairment resulting from TBI has led to enormous burdens to family and society [1]. According to the World Health Organization, TBI will surpass many diseases as a major health problem and leading cause of disability by the year 2020 [2]. After TBI, the subsequent development of mechanical injury or ischemia, hypoxia, ionic disequilibrium, and toxic effects of excitatory amino acids may damage or kill neurons or microvascular cells, leading to secondary edema, progressive hemorrhagic injury, and brain dysfunction.

Protecting neurons and microvascular cells from damage and death is important for rescuing neurological function. Cellular cell death or survival is determined by the integration of multiple survival and death signal pathways. The activation of phosphatidylinositol 3-kinase (PI3K) is correlated with increased cell survival, and this effect is largely mediated through the activation of a serine/threonine kinase Akt. The PI3K/Akt pathway promotes cellular survival in part by phosphorylating and inhibiting death-inducing proteins, including glycogen synthase kinase 3 (GSK-3), Bcl-2/Bcl-xL-associated death protein (BAD), and caspase- 9 [3]–[6].

Phosphatase and tensin homolog deleted on chromosome 10 (PTEN), a dual-specificity phosphatase, comprises an N-terminal phosphatase domain, a C2 domain, and a C-terminal tail domain that has a PDZ [Post synaptic density protein (PSD95), Drosophila disc large tumor suppressor (DlgA), and Zonula occludens-1 protein (ZO-1)] domain-binding sequence. The phosphatase domain specifically dephosphorylates the D3 inositol headgroup of phosphoinositol 3,4,5-triphosphate, leading to generation of phosphoinositol 4,5-bisphosphate [7], [8]. Through this domain, PTEN plays a key role in cell migration, survival, apoptosis, angiogenesis, and tumor formation by negatively regulating phosphoproteins in the PI3K/Akt pathway [9]–[13].

In this study, we investigated the role of PTEN in rats that underwent TBI induced by fluid percussion injury (FPI). We discuss the influence of bpV (pic) on neuronal death, blood brain barrier (BBB) permeability, and neurological function recovery.

## Materials and Methods

### Drug preparation, administration and FPI model of rats

A total of 169 rats were used in this study. We used a random number table for the randomization of the rats.

Animals were given bpV (pic) (Enzo, Farmingdale, NY, USA) at a dose of 20 µg/100 g four times at an interval of 3 h by intraperitoneal injection as previously described [14], and TBI was induced 15 min after the last injection. bpV (pic) was dissolved in 0.9% saline, and control rats received intraperitoneal injections of 0.9% isotonic saline without bpV (pic).

**Figure 1:**
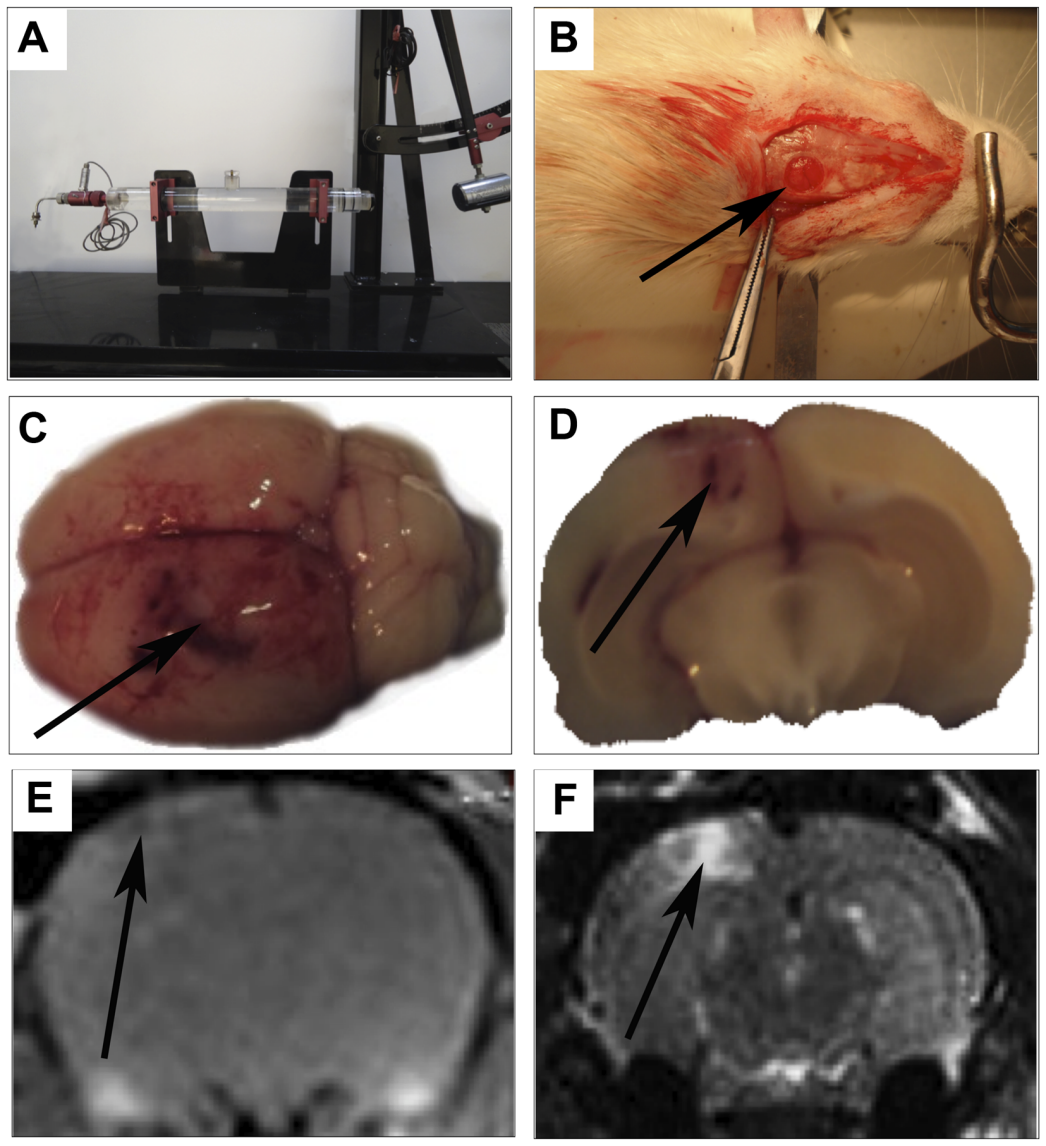
The process of FPI in rats. A: the FPI machine. B: injury site (arrow). C and D: brain tissue shows the injury core (arrows). E and F: MRI shows the injury core (arrows).

We used the unilateral rat FPI model in this study [15]. In brief, male Sprague-Dawley rats (250–300 g) were anesthetized with 4% chloral hydrate by intraperitoneal injection. The temperature was maintained at 37°C by a thermal heating pad. A craniotomy (approximately 4 mm in diameter) was performed at the right lateral skull, such that the medial edge of the craniotomy was approximately 2 mm from the midline suture, midway between the bregma and lambda. A polyethylene tube with an inner diameter of approximately 4 mm was fixed to the opening with cyanoacrylate adhesive and dental acrylic, filled with 0.9% isotonic saline, and attached to the FPI device. Rats were subjected to moderate extradural FPI with 2.1-atm injury. The duration of the waveform response due to fluid percussion was recorded as 12 to 15 ms. Then the scalp was sutured (Figure 1). Sham animals received the same surgical procedures except FPI. All the rats were returned to the temperature-controlled room via air conditioning and thermal heating pad. All of the surgical, injury, and animal care protocols described above were approved by the Scientific and Ethics Committee of Shanghai Jiaotong University.

### Tissue preparation

**Figure 2:**
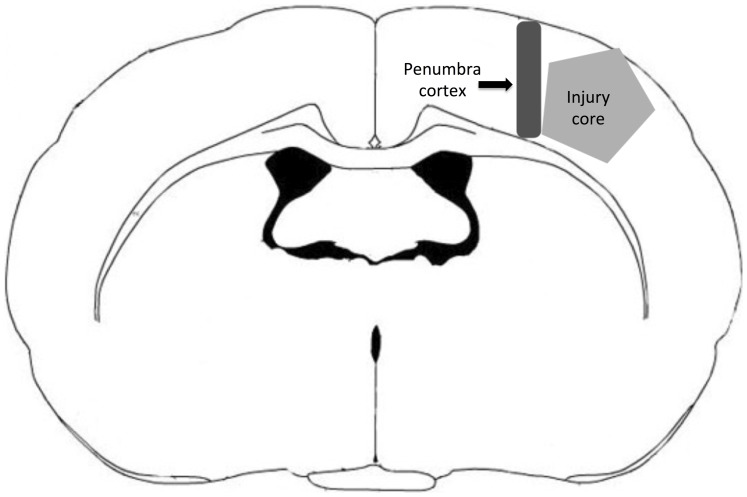
Anatomical location of the histological sections: penumbra cortex.

For brain tissue preparation, rats were sacrificed under anesthesia at different time points. Before sacrifice, the chest was opened and perfused with 0.9% saline through the left ventricle until colorless perfusion fluid was obtained from the right atrium. Whole brains were removed for dissection. An 8×8-mm area of cortex containing the injury core was removed. At 2, 6, 24, 48, and 72 h after TBI, brains from sham controls, the bpV (pic)-treated group, and the non-bpV (pic)-treated group were collected for experiments. Tissues used for western blotting and real-time quantitative PCR (Q-PCR) were stored in liquid nitrogen and then at −80°C. For the TUNEL test, rats were perfused with 4% paraformaldehyde in 0.1 M phosphate buffer after saline perfusion, embedded in paraffin, and stored at −80°C. For immunostaining, tissues were perfused with 4% paraformaldehyde following 0.9% saline, and then immersed in 30% saccharobiose. Tissues were stored at −80°C after being embedded in optimal cutting temperature compound (OCT). The anatomical location of the histological sections used in the analyses is the penumbra cortex (Figure 2).

### Western blotting

Whole protein extracts were obtained by homogenizing tissue samples in RIPA lysis buffer. Denatured protein extracts (30 µg) were electrophoresed on 10% SDS–PAGE gels and then transferred to PVDF membranes. Membranes were blocked with 5% non-fat milk and probed with PTEN (1∶1000) purchased from Abcam (Cambridge, MA, USA) as a primary antibody. After washing three times, membranes were probed with appropriate secondary antibodies (Abcam, Cambridge, MA, USA) including anti-rabbit IgG, HRP-linked antibody (1∶2000), or anti-mouse IgG, and HRP-linked antibody (1∶2000) for 1 h at room temperature. Membranes were washed, and immunopositive bands were visualized with a Kodak imaging system using a SuperSignal West Pico chemiluminescence kit (Pierce, Rockford, IL, USA). GAPDH was used as a loading control. The optical densities of protein bands were semi-quantified with Image J software.

p-Akt and MMP-9 were tested as described above. Anti-p-Akt was purchased from Cell Signaling (Danvers, MA, USA) and anti-MMP-9 were purchased from Abcam (Cambridge, MA, USA).

### Real-time Q-PCR

The Q-PCR assays were performed in a final volume of 20 µl ,which included 5 µl of the subjects' gDNA, as described by Marin et al [16]. All samples, including all standard curve dilutions, were assayed in triplicate using a default ABI Step one plus (Foster City, CA, USA) real-time PCR instrument. The reaction conditions were an initial enzyme activation step of 10 min at 95°C, followed by 50 cycles of 15 s at 95°C and 60 s at 60°C.

The primers used for Q-PCR were: PTEN forward primer: 5′CGTGCGGATAATGACAAG3′; PTEN reverse primer: 5′TTGATGGCTCCTCTACTG3′; GAPDH forward primer: 5′GTCGGTGTGAACGGATTTG3′; and GAPDH reverse primer: 5′ TCCCATTCTCAGCCTTGAC 3′. The results of quantifying PTEN transcripts were expressed as percentage ratios relative to GAPDH transcripts.

### Immunofluorescence double labeling

Immunofluorescence double labeling was performed as previously described [17]. Briefly, brain tissues were sectioned and prepared for staining as described above and then incubated with the following primary antibodies: rabbit anti-PTEN (1∶100; Abcam) and mouse anti-NeuN (1∶200; Merck Millipore), a marker for neurons. Sections were incubated with primary antibodies overnight at 4°C and washed three times for 10 min with phosphate-buffered saline (PBS) the following day. They were then incubated with Alexa 488-conjugated goat antibody to rabbit and Cy3-labeled donkey antibody to mouse (1∶200; Abcam). All antibodies were diluted in a solution comprising 10% normal goat serum and 1% Triton X-100 in PBS. Pre-immune serum was used as a control to confirm the specificity of the antibody. Images were obtained with a laser scanning confocal microscope (80i; Nikon, Japan).

### TUNEL assay

Terminal deoxynucleotidyltransferase-mediated UTP end labeling (TUNEL) assay was conducted using an in situ cell-death detection kit according to the manufacturer's instructions (POD kit DBA; Roche, Indianapolis, IN, USA). Briefly, brain tissues were embedded in paraffin and then sectioned for the TUNEL assay. The sections were xylene- and ethanol-treated for paraffin removal and dehydration, then incubated for 30 min at 37°C with proteinase K working solution (10–20 µg/ml in 10 mM Tris/HCl, pH 7.4–8). They were then washed twice with PBS. Next, the sections were treated with a TNLE reaction mixture at 37°C for 1 h and washed with PBS three times. Converter-POD was added, and the slides were incubated for 30 min at 37°C and then rinsed three times with PBS. Finally, DAB substrate (Sigma) was added, and the slides were incubated for 10 min at 20°C and washed three times with PBS. We analyzed the slides under a light microscope (CX41; Olympus, Japan).

### BBB integrity

The integrity of the BBB was investigated by measuring the extravasation of Evans blue (EB) dye in bpV (pic)-treated animals and controls (*n* = 5 in each group). EB dye is known to bind to serum albumin after injection and has been used as a tracer for serum albumin. EB dye (2% in saline, 10 ml/kg) was administered intraperitoneally 22 h after TBI. Two hours after EB dye injection (24 h after TBI), the chest was opened under anesthesia, and the animal was perfused as described above to remove the intravascularly localized dye. After decapitation, the brain was removed and dissected into left and right hemispheres, and the injured hemisphere was weighed. Samples were homogenized in 2.5 ml of PBS and mixed by vortexing for 2 min after the addition of 2.5 ml of 60% trichloroacetic acid to precipitate the protein. Samples were cooled and then centrifuged for 30 min at 1000× *g*. The supernatant was measured at 610 nm for absorbance of EB using a spectrophotometer. EB was calculated as µg/mg of brain tissue against a standard curve.

### Neurological function testing

Behavioral measurements were performed by two blinded evaluators. Ten rats in each group [bpV (-) group and bpV (+) group] were used for behavioral testing. Rats (*n* = 20) were observed for 30 days after the injury, and the neurological severity score (NSS) was assessed at 6 and 12 h and at 1, 2, 3, 7, 14, and 30 days after injury. The different tasks of the NSS are used to evaluate motor ability, balancing, and alertness of the rat. One point is awarded for failing to perform a particular task (Table 1). When a rat died, it was excluded from the NSS evaluation (and was not scored arbitrarily as 10) [18].

**Table 1:** Neurological severity score (NSS) for head-injured rat.

Task	NSS points
Presence of mono- or hemiparesis	1
Inability to walk on 3-cm-wide beam	1
Inability to walk on 2-cm-wide beam	1
Inability to walk on 1-cm-wide beam	1
Inability to balance on 0.5-cm-wide beam	1
Inability to balance on 0.5-cm-diameter round stick	1
Failure to exit 30-cm circle within 2 min	1
Inability to walk straight	1
Loss of startle reflex	1
Loss of seeking behavior	1
Maximum total	10

For each failed task the mouse receives 1 point. Maximum  = 10 (failure in all tasks), minimum  = 0 (success in all tasks).

### Statistical analysis

Data were analyzed using IBM SPSS Statistics 20 software. Two-group comparisons were analyzed by Student's *t*-test, chi-square test or a non-parametric test. Multiple-group comparisons were performed by analyses of variance (ANOVA) followed by post hoc analyses (LSD). Kaplan-Meier analysis was used for survival analysis. Differences were considered significant at *p*<0.05.

## Results

### PTEN expression increased after TBI, and bpV (pic)-treatment promoted p-Akt expression

**Figure 3:**
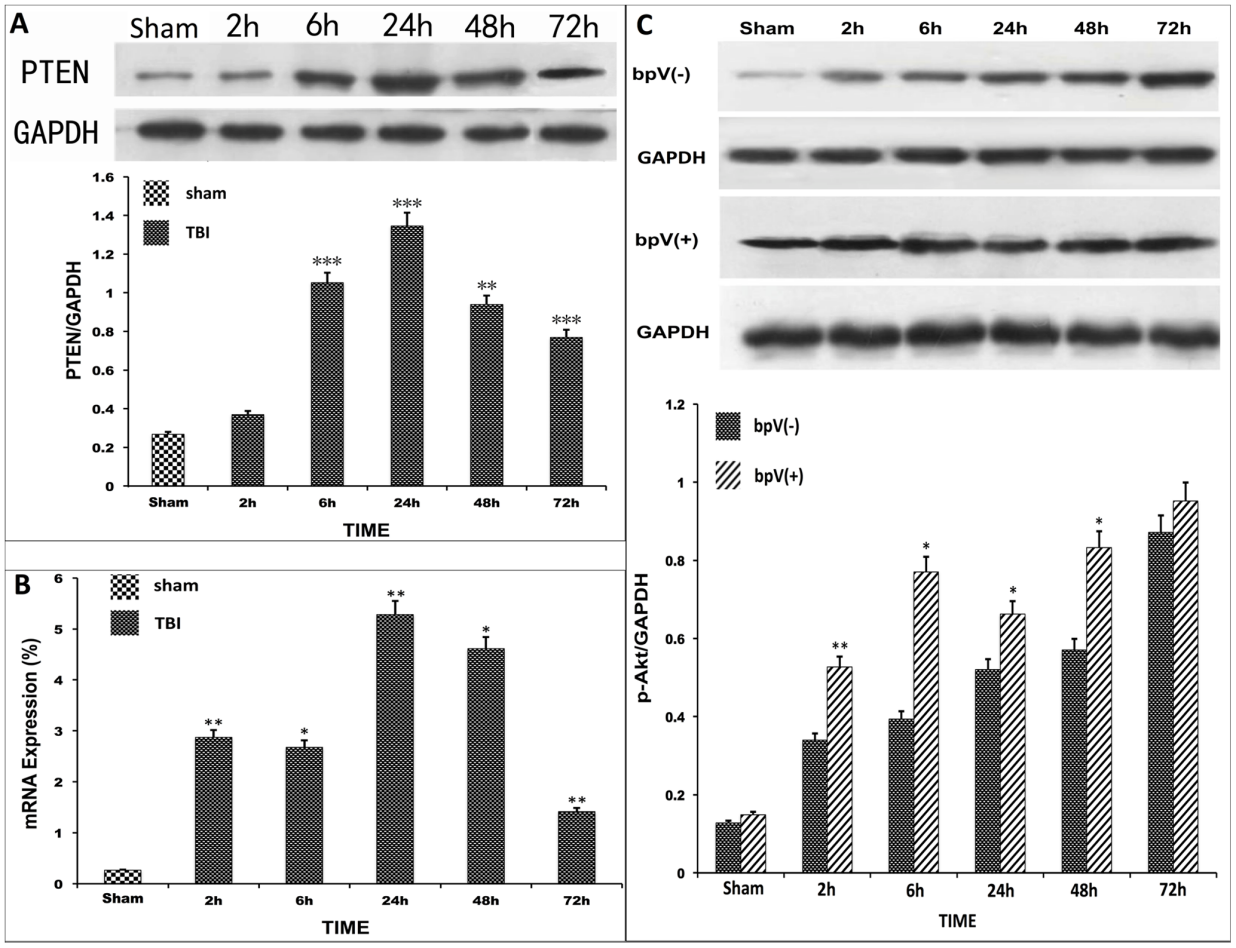
PTEN protein, mRNA and p-Akt protein expression after TBI. (A, B) PTEN protein and mRNA expression after TBI. PTEN expression was higher after TBI in the experimental than in the control group (compared with control, *p<0.05, **p<0.01, ***p<0.001). (C) p-Akt expression after TBI in the bpV (pic) (-)and bpV (pic) (+) groups. p-Akt was highly expressed after TBI. p-Akt was higher in the bpV (pic) (+) group than in the bpV (pic) (-) group [compared with bpV (pic) (-) group, *p<0.05, **p<0.01].

A total of 18 rats were used for testing PTEN expression (*n* = 3 in each group). Western blot analysis showed that TBI induced a significant increase in PTEN levels compared with controls. Early after injury, there was a marked increase in PTEN immunoreactivity. The increased immunoreactivity was sustained for more than 3 days, with a peak at 24 h; after 24 h, PTEN expression gradually decreased. However, after 72 h, the PTEN level remained significantly higher than that of controls (*p*<0.01) (Figure 3A).

Another 18 rats were used for determining PTEN mRNA levels (*n* = 3 in each group). Q-PCR showed that the PTEN mRNA level was about 10 fold that of the sham controls at an early stage, 2 h after TBI. In accordance with the PTEN protein, mRNA was also sustained for more than 3 days, with a peak at 24 h (*p*<0.05) (Figure 3B).

**Figure 4:**
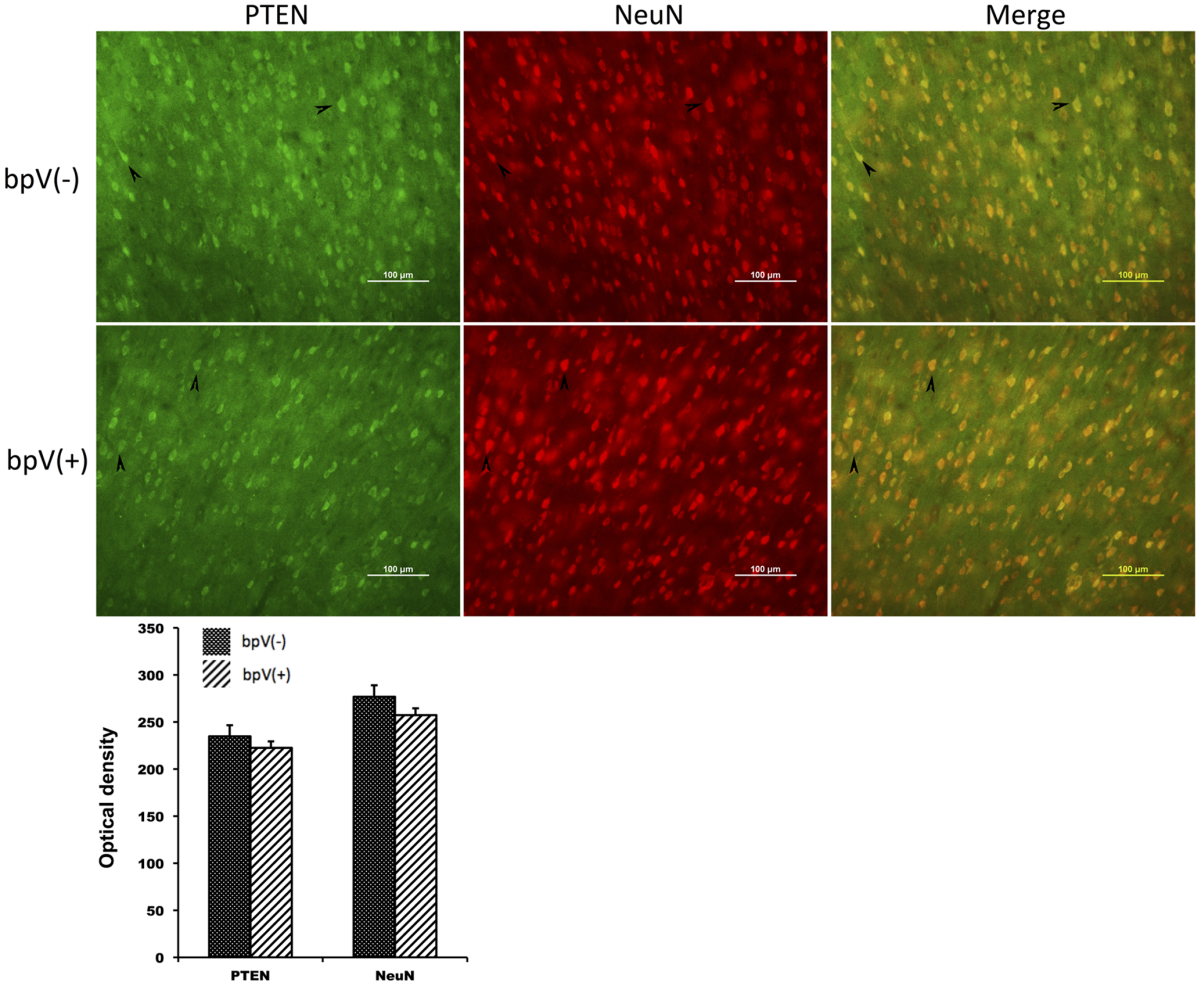
Representative images of the cortex 24-stained with the indicated antibodies. PTEN expression showed no significant changes after bpV (pic) treatment. Quantification of the optical density for both PTEN and NeuN immunoreactivity is shown on the panel at the bottom (*n* = 5 rats).

According to a previous report, bpV (pic) has no effect on PTEN expression [19]. Our results also showed no significant difference between the bpV (pic)-treated group and the non-bpV (pic)-treated group (Figure 4). Additionally, 18 rats were used for p-Akt testing (*n* = 3 in each group). Western blot analysis showed that the p-Akt level increased hourly after TBI, and p-Akt expression was significantly higher in the bpV (pic)-treated group than in the non-bpV (pic)-treated group at different time points (*p*<0.05) (Figure 3C). These results are consistent with those of a previous publication showing that bpV (pic) promotes p-Akt expression after ischemic brain injury [20].

### PTEN inhibition reduced neuronal apoptosis

**Figure 5:**
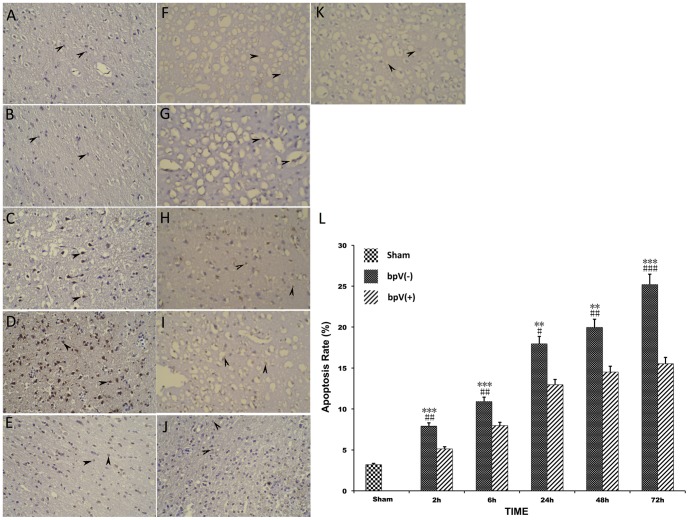
Neuronal apoptosis after TBI. (A–E), 2, 6, 24, 48, and 72 h after TBI without bpV (pic) treatment. (F–J), 2, 6, 24, 48, and 72 h after TBI with bpV (pic) treatment. (K) Sham control. (L) shows that neuronal apoptosis was significantly higher in the experimental in the control group after TBI. Cell apoptosis in the bpV (pic) (-) group was significantly higher than that in the bpV (pic) (+) group. [Compared with control, **p<0.01, ***p<0.001; compared with bpV (pic) (+) group, #*p*<0.05, ##*p*<0.01, ###*p*<0.001].

Inhibition of PTEN with bpV (pic) increased the expression of p-Akt, which should promote cell survival. We investigated whether apoptosis could be blocked with inhibition of PTEN. Neurons at 2, 6, 24, 48, and 72 h after TBI from both bpV (pic)-treated and non-bpV (pic)-treated rats were used to detect apoptosis using TUNEL assay (*n* = 5 in each group). We found that the number of TUNEL-positive cells increased hourly after TBI in bpV (pic)-treated compared with sham controls and persisted at a high level even after 72 h. Treatment with bpV (pic) reduced apoptosis at different time points compared with the non-bpV (pic)-treated group (*p*<0.01) (Figure 5).

### PTEN inhibition decreased BBB permeability

**Figure 6:**
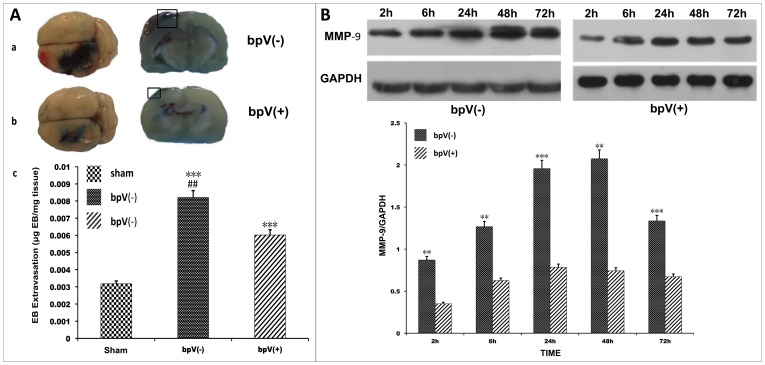
BBB permeability assay. (A) EB dye extravasation in bpV (pic) (-) group and bpV (pic) (+) group. a, b: rats were injected intraperitoneally with EB dye; pictures show brains of bpV (pic) (-) (a) and bpV (pic) (+) rats (b). In both groups, extravasation of EB dye into the parenchyma within the injured area indicates a permeable BBB. c: Photometric analysis of EB dye content in control and injured tissue. Enhanced absorbance was measured in the bpV (pic) (-)and bpV (pic) (+) groups than that of control; the EB content was higher in the bpV (pic) (-) group than in the bpV (pic) (+) group. [Compared with control, ***p<0.001; compared with bpV (pic) (+) group, ##*p*<0.01]]. (B) MMP-9 expression analysis at different time points via WB. Overexpressed MMP-9 was observed after TBI. bpV (pic) treatment decreased MMP-9 expression (compared with bpV (pic) (+), **p<0.01, ***p<0.001).

The EB dye in the samples taken from the TBI rats was higher than that in the samples taken from sham rats, revealing that TBI significantly increased BBB permeability (*p*<0.001). In the group that underwent TBI plus bpV (pic) (*n* = 5), BBB permeability was decreased compared with that in the group that underwent only TBI (*n* = 5) (*p*<0.01) (Figure 6A).

We also investigated MMP-9 expression using western blotting. MMP-9 is a biomarker of BBB disruption. MMP-9 disrupts the BBB by degrading the tight junction proteins and basal membrane proteins (e.g., fibronectin, laminin, collagen, and others), thereby leading to an increase in BBB permeability [21]. In this study, we found an over-expression of MMP-9 after TBI, and bpV (pic) decreased MMP-9 expression (p<0.01) (*n* = 30, *n* = 3 in each group) (Figure 6B). This indicates that PTEN inhibition via bpV (pic) may decrease BBB permeability.

### PTEN inhibition improved rat neurological function in the early stage after TBI but had no significant impact on mortality

**Figure 7:**
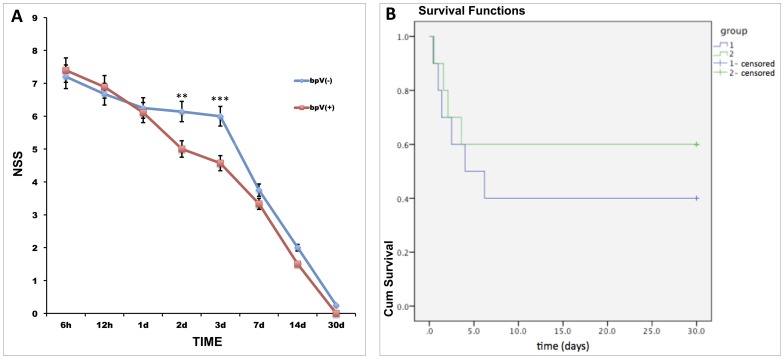
NSS and survival analysis of both groups. (A) NSS of both groups in the 30-day observational period. NSS was higher at 2 and 3 d after TBI in the bpV (pic) (+) group than in the bpV (pic) (-) group [compared with bpV (pic) (-) group, **p<0.01, ***p<0.001]. At other time points, the NSS showed no significant difference between the groups (*p*>0.05). (B) Survival analysis of 20 rats of both groups. The rats died within 7 days. At the end of the observation period, the mortality of the bpV (pic) (-) group was 60%, and that of the bpV (pic) (+) group was 40%. This difference was not statistically significant (*p*>0.05).

We used 20 rats for neurological testing: 10 in the bpV (pic)-treated group and 10 in the non-bpv (pic)-treated group. The NSS showed no significant difference at 6 h, 12 h, 1 d, 7 d, 14 d, and 30 d (*p*>0.05); however, the NSS was significantly different at 2 and 3 d (*p*<0.01) (Figure 7A). We focused on the two time points (2d and 3d) of interest and illustrate a point-by-point comparison. We found that there is significant difference between the two groups in exiting 30-cm circle within 2 min or not. More rats failed to exit the circle within 2 min in non-bpv (pic)-treated group than bpV (pic)-treated group, both in 2d and 3d (85.7% VS 0% and 60% VS 0%, respectively, p<0.05). In other aspects of NSS, no significant difference was observed. Such as, deficits in balance beam performance were common in both the groups, and no rat lost startle reflex (Table 2).

**Table 2:** NSS point-by-point comparison focused on 2d and 3d.

Task	non-bpv (pic)-treated	bpv (pic)-treated	*p*
	group (%)	group (%)	value
2^nd^ day			
Failure to exit 30-cm circle within 2 min	85.7	0	0.001
Inability to walk straight	57.1	25	0.315
Loss of seeking behavior	100	75	0.467
3^rd^ day			
Failure to exit 30-cm circle within 2 min	60	0	0.045
Inability to walk straight	40	0	0.152
Loss of seeking behavior	100	57.1	0.205

Other aspects of NSS have no difference between the two groups. We only listed the aspects which have difference between the two groups and the results of statistics.

At the end of the observation, we calculated the mortality of each group. Survival analysis showed that rats died in the early stage in both groups (most within 3 days). After 30 days of observation, six rats were alive in the bpV (pic)-treated group (mortality, 40%), but only four rats were alive in the non-bpV (pic)-treated group (mortality, 60%). However, the difference in the mortality rate between the two groups was not significant (*p*>0.05) (Figure 7B).

## Discussion

Neuronal apoptosis, BBB disruption, and neuronal function recovery are important issues after TBI. In this study, we found that (1) PTEN and p-Akt were overexpressed after TBI, (2) PTEN inhibition reduced neuronal apoptosis, (3) PTEN inhibition decreased BBB permeability and MMP-9 expression, and (4) PTEN inhibition improved neuronal function recovery in the early stage after TBI.

Apoptosis is a common pathological process after brain injury. Many factors impact the occurrence of apoptosis. Akt activation is well-established cascades downstream to PTEN inhibition, which contributes to axonal regrowth induced by PTEN deletion following neural injuries [22]–[24]. Akt exerts its role in promoting cell survival and growth by regulating the phosphorylation of its downstream components, including GSK-3, BAD, and caspase-9. Phosphorylation of serine (ser) at position 473 in Akt increases activity [25]. Our results show that p-Akt increased from 2 to 72 h after TBI. It is possible that injured neurons up-regulate p-Akt to facilitate recovery after TBI. This increase in the p-Akt level was also found in an ischemia model [26]–[28]. Furthermore, we found that treatment with bpV (pic) reduced apoptosis at different time points compared with the non-bpV (pic)-treated group. These results indicate that inhibition of PTEN via bpV (pic) reduced neuron death after TBI. The authors recently reported that excitotoxic NMDA stimulation increased PTEN nuclear translocation in cultured neurons, and PTEN nuclear translocation is an essential step leading to NMDAR-mediated neuronal death [29]. This may be a new cell-death mechanism involving PTEN. Besides reducing cell death, PTEN inhibition also promotes axon regeneration [30], [31].

After TBI, microvascular damage and BBB disruption occur. This promotes the development of brain edema and progression of hemorrhage and results in deterioration of the overall condition. Central nervous system neurovascular units are multicellular complexes comprising endothelial cells, pericytes, neurons, glial cells, growth factors, and extracellular matrix proteins in physical proximity to the endothelium [32], [33]. The generation of new vasculature facilitates highly coupled neurorestorative processes including neurogenesis and synaptogenesis, which in turn lead to improved functional recovery [34]–[36]. These two processes interact. We found that PTEN inhibition can decrease BBB permeability. This result is in accord with a previous study by Kini V, which showed that PTEN inhibition decreased endothelial permeability in human pulmonary arterial endothelial cells [12]. The authors reported that PTEN up-regulation prevented cell proliferation and migration and induced apoptosis of pulmonary artery smooth muscle cells under hypoxia, indicating that PTEN up-regulation may promote vascular damage [37], [38].

According to the above findings, PTEN inhibition prevents neuronal apoptosis and decreases BBB permeability. These changes contribute to post-traumatic neuronal functional recovery. We found no significant difference in NSS within 1 d or after 7 d after TBI, but a significant difference was observed at 2 and 3 d. In the very early stage (<1 d after TBI) and advanced stage (>7 d after TBI), PTEN inhibition has no impact on the NSS. This may because the neuronal function in the very early stage is associated with severity of the primary injury. In the advanced stage, neuronal function has reached an ideal status (NSS<3) and has changed only slightly over a long period, making differences in NSS hard to observe. From Figure 6A, we can see that NSS decreased by almost 4 points within 7 d, but by only 3 points during the 23-d subsequent period. Previous studies also reported that down-regulation of PTEN promoted neuronal function recovery, but the time point was different [27], [28]. This may because of the different evaluating standard (NSS vs. mNSS or other) or the different time points of observations.

The survival analysis revealed no significant difference in mortality at 30 d after TBI between the two groups and also showed that rats died within 7 d after TBI. However, the final mortality rates were 40% and 60% in the bpV (pic)-treated and non-bpV (pic)-treated groups, respectively. This is actually a substantial difference between the groups; however, maybe because of the small sample size (only 10 rats in each group), this difference is not statistically significant. No studies on mortality of TBI rats after bpV (pic) treatment have been reported.

The above discussion concerns PTEN in the central nervous system. PTEN inhibition is also implicated in intrinsic regenerative outgrowth of adult peripheral axons, regulation of axonal growth, and formation of neuromuscular junctions [21], [39].

Besides its function in the nervous system, as a tumor suppressor, PTEN affects breast cancer [40] and glioma [41]. It also plays a role in wound healing [42], leukemia therapy [43], and cardioprotection [44], [45]. Thus, PTEN is key point in many human diseases.

In this study, we investigated whether bpV (pic) treatment decreases neuronal apoptosis, BBB permeability, and evaluated its effect on neuronal function recovery. First, we showed that PTEN remained highly expressed for 72 h after TBI in the injured region of the brain. We then showed that in the injured brain, bpV (pic) increased activation of Akt, which has been shown to contribute post-injury neuroprotection in response to PTEN inhibition. Importantly, our results suggest that bpV (pic) decreases cell apoptosis and BBB permeability. To further demonstrate that PTEN inhibition has a neuroprotective function, we determined the NSS in bpV (pic)-treated rats and non-bpV (pic)-treated rats. Together, these data suggest that bpV (pic) decreases neuron death and BBB disruption via activation of Akt. However, whether PTEN overexpression increases neuronal death and BBB disruption requires further investigation.

## Conclusion

In conclusion, PTEN is a multifunctional protein that has a role in many diseases via complicated mechanisms. Our data suggest that treatment with the PTEN inhibitor bpV (pic) before TBI has a neuroprotective effect.
